# mSphere of Influence: the Value of Simplicity in Experiments and Solidarity among Lab Members

**DOI:** 10.1128/mSphere.00172-19

**Published:** 2019-06-19

**Authors:** Nicholas A. Wallace

**Affiliations:** a Division of Biology, Kansas State University, Manhattan, Kansas, USA

**Keywords:** DNA repair, cervical cancer, human papillomavirus

## Abstract

Nicholas Wallace studies how human papillomaviruses cause cancer throughout the genital and oropharyngeal tracts as well as in the skin. These viruses inhibit host DNA repair to promote their life style and in doing increase the risk of oncogenic mutations. In this mSphere of Influence article, he reflects on how two papers influenced him. “Human Papillomaviruses Activate the ATM DNA Damage Pathway for Viral Genome Amplification upon Differentiation” by C. A. Moody and L. Laimins (PLoS Pathog 5:e10000605, 2009, https://doi.org/10.1371/journal.ppat.1000605) reminded him of the power of straightforward approaches, while “Forty-Five Years of Cell-Cycle Genetics” by B. Reid et al. (B. J. Reid, J. G. Culotti, R. S. Nash, and J. R. Pringle, Mol Biol Cell 26:4307–4312, 2015, https://doi.org/10.1091/mbc.E14-10-1484) gave him the inspiration for his lab management style.

## Abstract (precis)

Nicholas Wallace studies how human papillomaviruses cause cancer throughout the genital and oropharyngeal tracts as well as in the skin. These viruses inhibit host DNA repair to promote their life style and in doing increase the risk of oncogenic mutations. In this mSphere of Influence article, he reflects on how two papers influenced him. “Human Papillomaviruses Activate the ATM DNA Damage Pathway for Viral Genome Amplification upon Differentiation” by C. A. Moody and L.

## COMMENTARY

Every scientist has their own unique approach, generated from a myriad of influences and personal experiences. Mentoring styles are even more individualized. Obviously, research mentors play an outsized role in shaping our approach to science. We are influenced by daily interactions with peers and lab mates, and every so often, there is a paper that changes the way we look at the world around us. The first time I read “Human Papillomaviruses Activate the ATM DNA Damage Pathway for Viral Genome Amplification upon Differentiation” by Cary Moody and Laimonis “Lou” Laimins is a vivid and influential memory (1). I had studied genomic instability induced by retrotransposons as a graduate student and had recently begun work as a postdoc in Denise Galloway’s group at the Fred Hutchinson Cancer Research Center (affectionately known as “The Hutch”). The question of how much skin cancer resulted from cutaneous human papillomavirus (HPV) infections fascinated me, but I was struggling to find my footing in this new field. My first ambitious project had plenty of technological bells and whistles. It had also just completely bombed. I felt left behind in the race to develop the fanciest approach to match the experiments that everyone else seemed to be running.

In the midst of these doldrums, Denise casually dropped Cary and Lou’s paper on my desk with a passing comment about it seeming like “something up your alley.” It was. Not only did the paper help me connect my graduate work to my new interests in viral oncology, it relied on elegantly designed experiments, rather than fancy, but hard to replicate approaches. The results were clear and definitive, relying on Southern and Western blotting combined with immunofluorescence microscopy to dissect HPV’s manipulation of DNA repair processes. Using familiar techniques, Cary and Lou had shown undeniable evidence that hyperactivation of a DNA repair signaling pathway facilitated HPV genome amplification. I felt hopeful again. Sure cutting edge techniques can drive a field forward, but there were clearly other paths to success. I poured my energy back into asking important questions and trying to answer them directly and precisely. The impact of Cary and Lou’s dissection of altered ATM signaling is easy to find in every paper from my lab. Perhaps more generally important, it is a steady reminder that flashiness is not a requirement for good science.

Several years later and at another transition point in my life, I encountered a different kind of article that was similarly impactful. It was not a primary research article or even a review. “Forty-Five Years of Cell-Cycle Genetics” by Brian Reid et al. is a retrospective commentary about the early years in Leland Hartwell’s lab that was published almost exactly 1 week after I started my first faculty job (2). I read it about 3 months later, when I was back visiting the Galloway lab over the winter holidays. Brian’s lab and office are just down the hall from Denise’s at the Hutch, so we had spoken each day for years. It must have surprised him to see me back to my morning routine when I greeted him. I remember being filled with excitement from my new position, but beginning to realize how much I was unprepared for. I peppered him with questions, “Who should I hire?” “What should I do first?” “How do I attract students?” “How can I help undergraduate students do ‘good’ science?” and on and on. When I paused to breathe, Brian matched my enthusiasm with stories of his time as a student in the Hartwell lab. It was wonderful to see that the memory of 40 years earlier still energized him.

Before I left, he sent me a copy of the article that he and his contemporaries in the Hartwell lab penned about their early days together. It should be required reading for any new principal investigator, especially if they plan to interact with undergraduates. I would love for my students to have half as positive of an experience as Brian and his colleagues had. The article describes the perfect atmosphere for science and is filled with motivational quotes like “although the group was not large, it never seemed small, in part because interaction among the lab members was so constant and intense”. Who would not want to work in that environment?!? Life as a scientist has peaks of exhilaration, but it is also brutal. Surviving negative reviews, rejections, misbehaving experiments, and unexplained contaminations are a daunting task that we all face. Your lab environment can make all the difference. I model my lab atmosphere after the one described in “Forty-Five Years of Cell-Cycle Genetics”. It is the first paper people read when they join our team. “An atmosphere of total openness and high collegiality, in which no one worried about who would get credit for a particular idea” sounds like science utopia. The camaraderie that Brian Reid, Joseph Culotti, Robert Nash, and John Pringle describe should be the goal of every leader.
